# Baseline Modelling and Composite Representation of Unobtrusively (IoT) Sensed Behaviour Changes Related to Urban Physical Well-Being

**DOI:** 10.1007/978-3-030-51517-1_13

**Published:** 2020-05-31

**Authors:** Vladimir Urošević, Marina Andrić, José A. Pagán

**Affiliations:** 8 Digital Research Centre of Sfax, Sfax, Tunisia; 9 Institut Mines-Télécom, CNRS, Paris, France; 10 Université de Sherbrooke, Sherbrooke, QC Canada; 11 Digital Research Centre of Sfax, Sfax, Tunisia; 12 University of Sfax, Sfax, Tunisia; 13 Belit d.o.o. Beograd, Trg Nikole Pašića 9, 11000 Belgrade, Serbia; 14 The New York Academy of Medicine, 1216 Fifth Avenue, New York, NY 10029 USA

**Keywords:** Behaviour recognition, Wearable devices, Unobtrusive sensing, Well-being, Vital health parameters, Data labelling, Composite index modelling

## Abstract

We present the grounding approach, deployment and preliminary validation of the elementary devised model of physical well-being in urban environments, summarizing the heterogeneous personal Big Data (on physical activity/exercise, walking, cardio-respiratory fitness, quality of sleep and related lifestyle and health habits and status, continuously collected for over a year mainly through wearable IoT devices and survey instruments in 7 global testbed cities) into 5 composite domain indicators/indexes convenient for interpretation and use in predictive public health and preventive interventions. The approach is based on systematized comprehensive domain knowledge implemented through range/threshold-based rules from institutional and study recommendations, combined with statistical methods, and will serve as a representative and performance benchmark for evolution and evaluation of more complex and advanced well-being models for the aimed predictive analytics (incorporating machine learning methods) in subsequent development underway.

## Introduction

The urban public health, well-being monitoring, and prevention are recently being transformed from reactive to a predictive and eventually long-term risk mitigating systems, through a number of research initiatives and projects, such as the ongoing PULSE Project (**P**articipative **U**rban **L**iving in **S**ustainable **E**nvironments, funded from the EU Horizon 2020 programme) focusing on the chronic metabolic and respiratory diseases (such as type 2 diabetes and asthma) affected or exacerbated by the preventable or modifiable environmental and lifestyle factors, and well-being/resilience. A major challenge in the Project is the modelling and assessment/prediction of citizen well-being from the collected and processed Big Data of unprecedented variety and from highly heterogeneous sources (health and vital activity personal data obtained through wearable devices and other sensing technologies, geo-located online surveys, open/public smart city datasets…), on individual and collective (population/cluster) levels. Overall well-being and its main domains (vitality, supportive relationships, stress levels…) are all significant factors affecting the onset and exacerbation of the stated chronic diseases which are becoming more and more widespread and progressing in urban environments, and overall resilience of citizens and urban communities is increasingly important against other pertaining global and sustainability challenges, like climate change.

The proposed and deployed elementary statistical model presented in this paper is to be the basis for interpretation and contextualization of changes to well-being, and a performance benchmark for evaluation and comparison of more complex and advanced well-being models of the aimed predictive analytics and final intelligent system (incorporating machine learning methods) in subsequent development, supporting the PULSE PHOs (Public Health Observatories established for the relevant policy making and execution in smart cities).

## Conceptual Background and Approach

The activity and vital/health parameters data measured mostly unobtrusively by wearable devices (wristbands, smartwatches) have particular significance for behaviour analysis and change recognition in PULSE, as these are the input data streams with highest volume, acquisition “velocity”, and temporal resolution/granularity of all the various data collected in the Project, and therefore practically the most (and only) suitable data comprising the sufficiently continuous and non-sparse time series over months, to properly derive or construct the behavioural patterns and analyze behaviour changes. Recent studies performed by the stated major wearable device manufacturers over billions of records of temporal measurements data [1, 2], as well as the experiences from projects like the just concluded City4Age (www.city4ageproject.eu) [3, 4], show the significance and general predictive ability of the measured main vital/health and activity parameters (walking, climbing stairs, physical activity/exercise, heart rate data, consumed calories…) for overall health and physiological/physical well-being assessment. The additional complementary socio-demographic, health, lifestyle/habits and environmental data in less frequent temporal resolution, ingested from the open/public datasets or manual “obtrusive” inputs, are combined to cross-check, adjust and improve integrity of the recognized behaviour changes derived from the main time-series data acquired through the wearable devices.

We adopt a combined knowledge- and data-driven approach in detection and characterization of relevant behaviours that denote significant variations in well-being, with multi-level hierarchical model topology and range/threshold based computational rules as basic primary formal knowledge structures, and statistical analytics as baseline (and performant) data-driven detection methods.

The complexity of human behaviours is commonly represented through multi-level hierarchical structured models, decomposed to more granular “units” like activities and action events [5, 6], with multiple variables from behavioural, physiological and environmental domains of well-being known to additionally increase complexity and dimensionality [7]. There are other contending approaches, like the monitoring and analysis of individual well-being or behavioural domain indicators or determinants independently in parallel, without hierarchical structuring and substantial synthesis into fewer higher-level composite factors or score(s) [8]. Most, including the adopted and followed approach works (like [9]), are comprehensively covered or referenced in the *Encyclopedia of Quality of Life and Well*-*Being Research* (Springer 2014) that summarizes recent research works related to well-being and quality of life in spanned various research and policy-making/implementation fields. Main advantages of a few composite synthetic indicators/factors over a battery of multiple separate indicators, namely:

- ability to summarize complex and multi-dimensional real-life phenomena or domains (like well-being),
- easier for interpretation and comparison among (socio-demographic, geospatial/regional…) groups or population clusters,
- more effective for comprehending overall trends, particularly when a number of the underlying indicators denote opposing-trend changes,

are of crucial significance in usage and context settings of the PULSE project, with over 60 indicators formalized in the initial knowledge-based well-being model topology from the systematization of collected data, and with

- visible set of indicators to various stakeholders (policy makers, researchers, general public) needing to be minimal without omitting important underlying information,
- and collaboration, communication and comparison of complex dimensions by various stakeholders needing to be most straightforward, facilitated and effective.

**Fig. 1.**
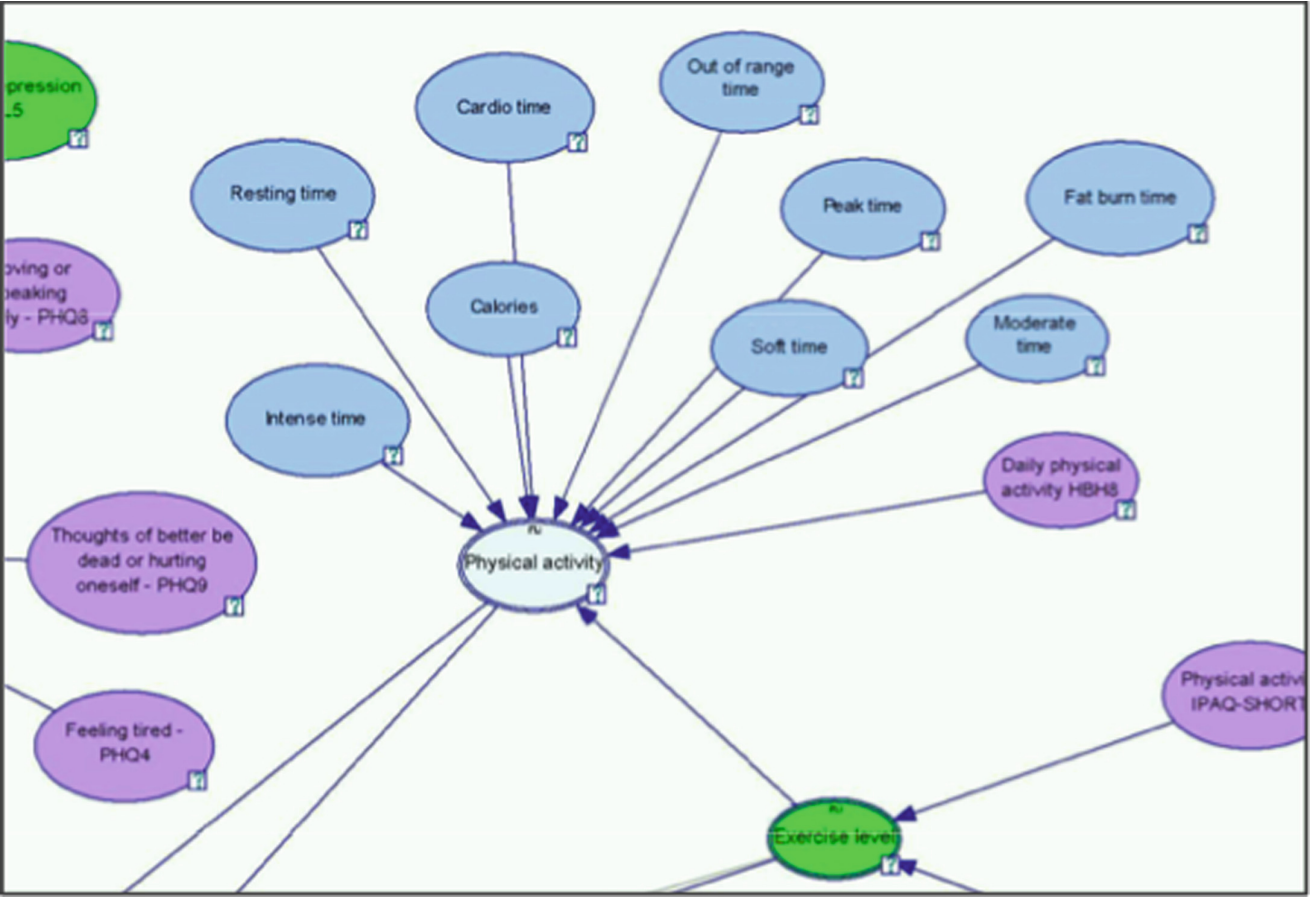
Excerpt from the hierarchical network structure topology of the initial knowledge-based PULSE well-being model (*blue*-*coloured nodes* are variables measured by wearable sensing devices, and *magenta*-*coloured* are reported/input through online app. questionnaires) (Color figure online)

We therefore propose two complementary approaches for synthesis of the composite well-being indicators composed from underlying streamed IoT-sourced time-series data in the context of PULSE. The indicators summarize multi-dimensional aspects of citizen well-being and enable the assessment of individual and synthesized collective urban well-being over time. The notion is illustrated through analysis within the scope of four representative and characteristical key summary indicators of citizen health and fitness, derived from activity and vital/health parameters measured, as stated, using wearable sensing devices: motility, physical activity, sleep quality and cardio-respiratory health/fitness (Fig. 1).

In the first approach, daily and intra-daily underlying measurements (Table 1) are used to estimate levels of adherence to rule- and range-based recommendations matured from institutional knowledge of relevant authorities and population-significant studies in the field, accumulated for over decades in the stated four example domains of motility, physical activity, sleep quality and cardio-respiratory fitness [8, 10, 11].

**Table 1.** List of variables measured by the used wearable devices, with descriptions and *units of measure* (SI base units in square brackets where applicable, like [*s*]-*seconds*, or [*m*]-*meters*)

Measured variable	Description [unit]
still_calories	amount of calories burned while being still
total_calories	total amount of calories burned (default in a day)
walk_steps	number of walked steps
walk_distance	walked distance [m]
stairs_floor_changes_up	number of floors traversed in climbing stairs up
elevation	vertical distance [m] traversed in climbing stairs
still_time	time [s] spent being still
physicalactivity_soft_time	time [s] spent performing light/”soft” physical activities like relaxed walking/strolling or standing up and moving around in the home, workplace or community
physicalactivity_moderate_time	time [s] spent performing moderate physical activities like swift walking, dancing, gardening
physicalactivity_intense_time	time [s] spent performing intense/vigorous physical activities like running, fast cycling or swimming, tennis, jumping rope
physicalactivity_calories	amount of calories burned while performing physical activities
heartrate_cardio_time	time [s] duration of the heart rate being within the defined “cardio” target range
heartrate_peak_time	time [s] duration of the heart rate being within the defined “peak” target range
heartrate_resting	resting heart rate
heartrate_avg	average heart rate
heartrate_max	maximal heart rate
sleep_time	total time [s] spent in all phases of sleep
sleep_efficiency	calculated score derived from sleep_time, sleep_asleep_time and sleep_wake_time
sleep_deep_count	number of times falling into deep sleep
sleep_deep_time	total time [s] spent in deep sleep
sleep_light_count	number of times falling into light sleep
sleep_light_time	total time [s] spent in light sleep
sleep_rem_count	number of times falling into REM sleep stage
sleep_rem_time	total time [s] spent in REM sleep stage
sleep_wake_count	number of times waking up
sleep_wake_time	total time [s] spent in waking up
sleep_awake_time	total time [s] spent awake during sleep
sleep_awake_count	number of times awakening during sleep
sleep_restless_time	total time [s] spent being restless during sleep
sleep_restless_count	number of times getting restless during sleep
sleep_asleep_time	total time [s] spent being fully asleep
sleep_asleep_count	number of times falling fully asleep

The complementary data-driven statistical approach is predicated on standard scores that denote the number of standard deviations that a given measurement deviates from the sample mean. This approach allows comparison of individual scores to the corresponding norm groups stratified by common socio-demographic parameters (age, gender…), when considered conditionally independent nodes in the complete model topology. It also allows to place a score for any individual and variable with respect to alternative descriptive statistic or measure of central tendency (variable median, geometric mean, standard deviation or error), so that more accurate or optimal comparison for specific variable distribution can be made.

## Citizen Activity and Vital/Health Parameters Data

The data are collected by several types of health and fitness wearable tracker devices manufactured by Fitbit, Garmin and ASUS, monitoring physical and walking activity, sleep and heart/cardio parameters, for over 300 recruited citizens participating in the study in 7 global testbed cities (Barcelona, Birmingham, New York, Paris, Pavia(Italy), Singapore, and Keelung/Taipei), supplied with wearable tracker devices by the Project.

Physical activity level as a single measure is mainly expressed in terms of time spent and calories burned while performing light/soft, moderate, and intense/vigorous physical activity. Walking activity is captured with walked steps, distance, speed, and climbed stairs/floors measurements. Heart rate measures capture time spent in different target heart rate zones (like peak, cardio, fat burn), resting and maximal heart rate (*hr*_*max*_), and some still experimental measures like systolic and diastolic blood pressure, measured by the newest recently released devices such as ASUS VivoWatch BP, but not yet acquired in significant volume sufficient for analysis. The peak heart rate zone by default definition ranges from 85 to 100% of person’s maximum heart rate (*hr*_*max*_), the cardio zone ranges from 70 to 84% of *hr*_*max*_, and the fat burn zone ranges from 50 to 69% of *hr*_*max*_. Sleep quality/hygiene measures mainly capture time spent in defined phases of sleep. All the processed measures are listed in Table 1, collected or aggregated with a default daily periodicity, except the ones in gray-shaded rows which are acquired in higher intra-daily temporal resolution, mostly once in every 15 min or up to once in a minute, depending on the variable.

In addition to stated behavioral time-series data, an extensive set of personal socio-demographic, profile (age, gender, ethnicity, educational and marital status, employment status and occupational environment…), as well as health state, risk factors and habits, lifestyle, neighbourhood and quality of life assessment, and other relevant behavioural data are manually input/submitted on each citizen participating in the study via online forms, composed from adapted relevant survey/assessment instruments for each specific field, like Framingham, EuroQoL-5, IPAQ-SF. These data are geo-localized to the residence location of each responding citizen for the purposes of analytics of collective/community well-being, and are collected from a greater number of recruited respondents, but just in rare cases in more than one iteration over time due to the high number and scope of covered variables, and therefore suitable for a broad but mainly static “snapshot” assessment of current well-being state rather than for behaviour change model and analytics. Incorporating both these static and IoT-sensed temporal data into a fully comprehensive predictive well-being model is an ongoing task in progress throughout the end of the Project, with results to be presented in other upcoming publications.

## Derivation of Domain/Dimension Indices

### Physical Activity/Exercise

Physical activity in our first approach stated above in Sect. 2 can be discretized using several common baseline categorizations related or derived from the above mentioned relevant institutional/governmental and professional expert guidelines for the urban population groups. The example approach taken in the recent health survey of England from 2016 [12] compared well-being and mental health of adults in different socio-demographically stratified population groups by physical activity, among others. The activity level categories used in the analysis were the following:

- *Meets aerobic guidelines*: At least 150 min moderately intensive physical activity or 75 min vigorous activity per week or an equivalent combination of these
- *Asserted activity*: 60 to 149 min moderate activity or 30–74 min vigorous activity per week or an equivalent combination of these
- *Low activity*: 30 to 59 min moderate activity or 15 to 29 min vigorous activity per week or an equivalent combination of these
- *Inactive*: Less than 30 min moderate activity or less than 15 min vigorous activity per week or an equivalent combination of these,

and the corresponding linear scaled scoring function denotes “*Meets aerobic guidelines*” with a score of 4, “*Certain activity*” - 3, “*Low activity*” - 2, and “*Inactive*” with 1. This baseline scoring scale, besides sufficient granularity and robustness exhibited in referenced comprehensive studies, is also convenient for

- mapping to the defined activity level categories used as input parameters for the consensus models for prediction of risk of Type 2 Diabetes (T2D) and asthma onset and exacerbation, developed for the PULSE project [13, 17]
- quantification of longer-term and/or periodic activity level behaviours directly from categorized daily or incidental activity level values as measured and acquired from the wearable tracker devices through relevant APIs (*light/soft*, *moderate*, and *vigorous/intense* activity).

### Specific Walking Activity

Similarly, the authors in [14] and [15] demonstrate the following referent threshold ranges of the number of daily walked steps to be used for classification of walking activity in healthy adults, and the corresponding scoring function linearly assigning the following 1–5 integer scores to the classification categories: *highly active* (12,500 or more steps/day) – 5, *active* (10,000–12,499 steps/day) – 4, *somewhat active* (7,500–9,999 steps/day) – 3, *lowly active* (5,000–7,499 steps/day) – 2, and *sedentary* (under 5,000 steps/day) - 1.

### Cardiovascular Fitness - VO_2_max

VO_2_max is the metric denoting the maximum amount of oxygen that an individual can use during intense exercise. It is widely and commonly used as an indicator of cardiorespiratory fitness.

A simple generic estimate of VO_2_max of an individual can be obtained using their maximum and resting heart rates in the following formula, publised in [16]:

1 $$ VO_{2} \hbox{max} \approx \frac{{hr_{max} }}{{hr_{rest} }} * 15.3  \,mL/(kg * min) $$

Where $hr_{max}$ can be crudely estimated as 220 – *age of the person*.

A relatively standard convenient and meaningful categorization of VO_2_max for Western European and USA populations can be on a scale from 1 - *very low*, through 2 - *low*, 3 - *fair*, 4 - *moderate*, 5 - *good*, and 6 - *very good*, to maximal 7 - *elite*, depending of the individual’s gender and age, with common categorization for males and females aged 6 to 75 published by Shvartz & Reibold in [11].

### Quality of Sleep

Total average sleep duration in 24 h is a straightforward direct metric for assessing the quality of sleep in terms of longer-term stable behaviour across complete populations. The US National Sleep Foundation recently provided the following referent expert sleep duration recommendations (in terms of recommended (or not) threshold values for both oversleep and undersleep), categorized by precise granular age ranges [10]:

These recommendations categorize possible output sleep duration times as either *recommended*, *may be appropriate*, or *not recommended*, and the optimal *recommended* duration is 7–9 h for majority of the populations.

Additionally, relevant recent findings like the extensive meta-analysis performed by the American Diabetes Association to assess the dose-response relationship between sleep duration and risk of type 2 diabetes [18], have concluded that the lowest type 2 diabetes risk is for the average overnight sleep duration from 7 to under 9 h per day, and that both shorter and longer sleep durations than this optimum range denote up to 1.5 times increased risk (and up to 2 times increased cardiac conditions risk shown in the related studies [21], also relevant in the Project). We therefore slightly alter the *may be appropriate* category from the otherwise adopted recommendations from Table 2 above to *mildly risky*, reflecting the importance of stated health risks in PULSE, and the effect of common or periodically repeated behaviour patterns over months or years to the exasperation of the risks. This categorization will also consequently be communicated on the data visualizations and public health/prevention interventions and campaigns deployed and administered through relevant PULSE system applications and modules (PHO Dashboards, PulsAIR gamified mobile app.) towards the citizens and urban communities, and the resulting function scores assigned to the categories are therefore: **1** for *not recommended*, **1.75** for *mildly risky*, and **2** for *recommended*, inversely proportional to the pesimistically estimated risks increase brought by shorter durations. Complex eventual relations of detailed specific measured sleep parameters to well-being will be explored by more advanced methods in other subsequent work.

**Table 2.** Detailed US National Sleep Foundation recommended threshold values and ranges for sleep duration per age category

Category *age*	Recommended	Considered appropriate	Not recommended
Newborns *0*–*3 months*	14 to 17 h	11 to 13 h 18 to 19 h	Less than 11 h More than 19 h
Infants *4*–*11 months*	12 to 15 h	10 to 11 h 16 to 18 h	Less than 10 h More than 18 h
Toddlers *1*–*2 years*	11 to 14 h	9 to 10 h 15 to 16 h	Less than 9 h More than 16 h
Preschoolers *3*–*5 years*	10 to 13 h	8 to 9 h 14 h	Less than 8 h More than 14 h
School-aged Children *6*–*13 years*	9 to 11 h	7 to 8 h 12 h	Less than 7 h More than 12 h
Teenagers *14*–*17 years*	8 to 10 h	7 h 11 h	Less than 7 h More than 11 h
Young Adults *18*–*25 years*	7 to 9 h	6 h 10 to 11 h	Less than 6 h More than 11 h
Adults *26*–*64 years*	7 to 9 h	6 h 10 h	Less than 6 h More than 10 h
Older Adults *65 years and over*	7 to 8 h	5 to 6 h 9 h	Less than 5 h More than 9 h

### The Composite Physical Well-Being Indicator

From several existing elementary statistical approaches for aggregating the underlying dimensional indices and constructing the summary composite indicator value, we consider the weighted geometric mean of the four constituting dimensional indices as most adequate and appropriate for this specific well-being problematics:

2 $$ WB_{ph} = \sqrt[4]{{I_{w}^{{Wt_{w} }} *I_{p}^{Wtp} *I_{s}^{{Wt_{s} }} *I_{c}^{{Wt_{c} }} }} , $$

where summary dimensional indices denoted by the scoring function values are:

$I_{w}$ – walking activity index, $I_{p}$ – physical activity/exercise index, $I_{s}$ – sleep duration index, $I_{c}$ – cardio-respiratory fitness index (through VO_2_max), and $Wt_{w}$, $Wt_{p}$, $Wt_{s}$, and $Wt_{c}$ are respective weight factors, derived from expert assessments and rank data from relevant previous studies and experience, and assigned to adjust the relative importance and contribution of each of the indices to the resulting composite indicator value, per compositing methods outlined in [19] and [9], or for derivation of composite UN Human Development Index (HDI). As all 4 constituting indices are directly proportional to the resulting composite indicator (i.e. the higher the activity levels or cardio-respiratory fitness scores, the higher the well-being), and low value of either of the four is significant for decreased overall composite (although there is some correlation between the indices - e.g. decrease in cardio-respiratory fitness in most cases causes decreased activity levels as well), the geometric mean is adequate for its sensitivity to low values of each individual constituting index, and ability to combine values on completely different scales without normalization required. Initially assigned values of weight factors are 0.9 for $I_{w}$, 1 for $I_{p}$, and 1.05 for $I_{s}$ and $I_{c}$, taking into account the importance of specific indices for respiratory disease and T2D risk, volatility of the collected data by now, and known overestimation of some measured variable values (like number of walked steps, VO_2_max estimate, or recognized sessions of cycling and some other exercise types) by the predominantly used wearable devices - Fitbit Charge 2 [20]. The weight factors are set as configuration parameters in the model, so they can be changed to fine-tune the composition according to the data insights acquired over time or the results of the validation described in Sect. 5 below.

Time series of the values of the composite indicator are formed from weekly and monthly aggregations of underlying daily and intra-daily measurements into the 4 constituting index values. Method for computing those values from the measured values of variables listed in Table 1 above is as developed and introduced in [22] for synthesis of indicators and geriatric factors from the same source IoT data, based in this case on univariate normalization of relative changes (quantified in standard scores, as stated above in the “Approach” Sect. 2) of acquired Big temporal Data during the complete study period, and then multivariate weighted linear aggregation of obtained normalized indicators and descriptive statistics into higher-level composite factors, to capture weekly and monthly behavioural patterns and trends, less susceptible to influence of outliers and ocassional notably deviating values.

## Preliminary Validation of the Composite Index and Conclusion

Validation metric is the correlation with specific corresponding summary measure(s) of current well-being, self-reported by the respondent citizens through web and mobile app. questionnaires as mentioned above. They can be summarized from two relevant subset questionnaires: 1) European Social Survey (ESS), and 2) EuroQoL-5D (EQ5) survey instrument, both standardized (with minor adaptations) and common for measuring well-being in multiple continuous and/or repeated relevant Europe-wide and national-level studies, and robust to some degree against extreme fully subjective bias.

15 statements of the ESS questionnaire broadly cover social and most of the other aspects of personal and community well-being that the respondent rates on a 5-degree Likert scale (*Strongly agree*-4, *Agree*-3, *Neither agree nor disagree*-2, *Disagree*-1, *Strongly disagree*-0), the total possible questionnaire score thus ranging from 0, denoting the lowest/worst well-being, to 60 representing the optimum.

EQ5 instrument is focused on physical and mental health status and daily life activities measured in 5 dimensions (mobility, self-care, usual activities, pain/discomfort and anxiety/depression), also self-rated on a 5-degree scale from perceived worst to best like in ESS. Last question asks for assessment of the respondent’s overall health state (*hsa*) on the current day, on the scale from 0 (worst) to 100 (best imaginable), also mapped to a number ranged from 1 to 5 by the formula *1 *+* 4* * *hsa/100* for the purpose of this evaluation. Total cumulative EQ5 score thus ranges from 6 to 30.

**Fig. 2.**
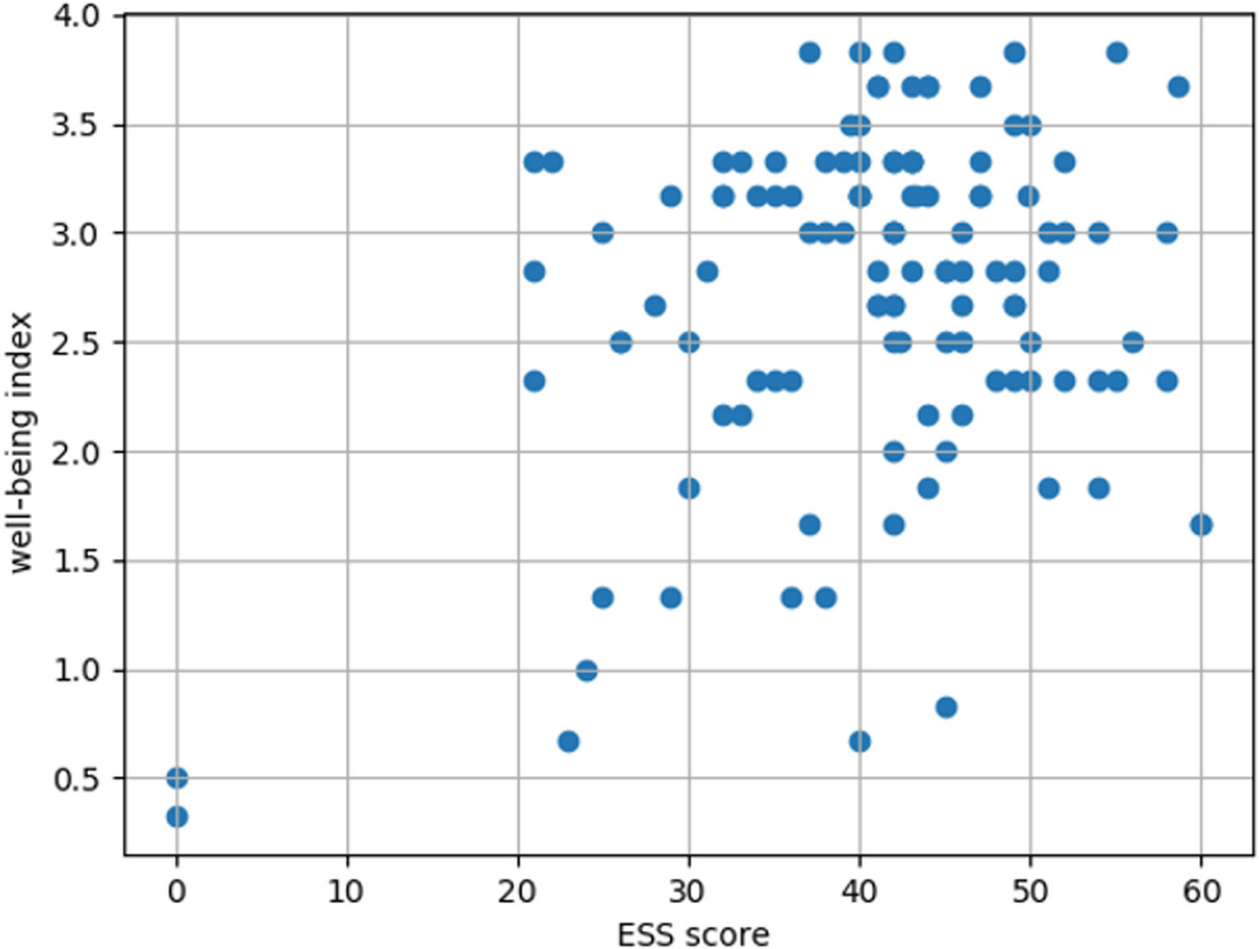
Scatter plot of composite well-being index vs. cumulative ESS score values

Figure 2 below shows the correlation scatter plot of ESS scores and composite well-being indices for 97 respondents of which 12 filled EES questionnaire twice, two filled it three times, and the rest only once during the observed period of 11 months.

**Fig. 3.**
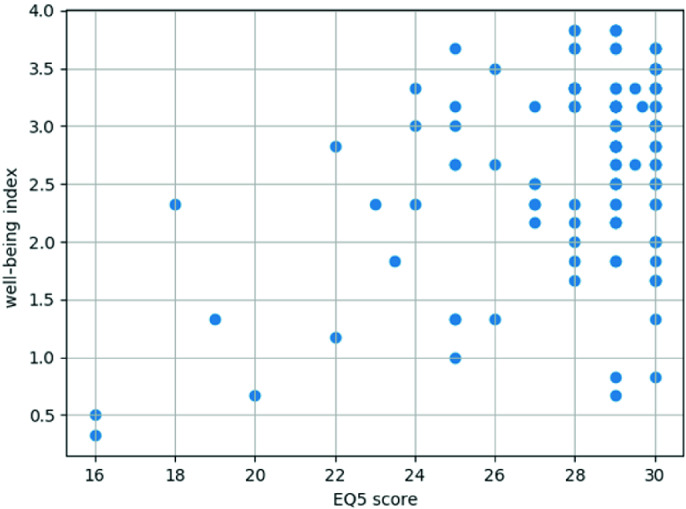
Scatter plot of composite well-being index vs. cumulative EQ5 score values

Figure 3 shows the relationship between the composite well-being indices and the obtained EQ5 scores of 107 respondents, 3 of which filled the questionnaire three times, 20 filled it twice, and the rest once during the observed data collection period.

The analysis reveals a medium positive correlation of 0.424 (with the p-value of approx. 3.7 × 10^−7^) between our constructed composite index and cumulative EQ5 scores. The composite indicator and its constituting domain indices can therefore be considered promising for their intended purpose of basic represention of the urban physical well-being aspects modelled from a variety of heterogeneous underlying activity and health/vital parameters measured by IoT wearable devices, summarized in 4 main dimensional and one overall derived score convenient for comparisons, interpretation and presentation to the end-users, particularly in the required shortest most concise manner and form, such as through a mobile app. UI or intervention messages.

As almost half of the questions in EQ5 are very remotely or not at all related to the physical well-being aspects summarized by the composite indicator, the correlation is expected to increase when the ongoing work in incorporating social and other well-beung aspects fully in the model is completed.

Found small positive correlation of 0.287 (p-value 0.001) between the composite indicator and cumulative scores of ESS questionnaire (in which most of the questions are not related to physical well-being) additionally points to the significance of this composite indicator to the overall well-being. The work also continues on the cleaning and pre-processing the data collected on the remaining monitored citizens, incorporation of machine learning methods in the model and exploration and modelling of the influence of detailed sleep and cardiac parameters, as well as of the sensed ambiental data, on the main well-being domains.
